# A Simple, Safe and Efficient Synthesis of Tyrian Purple (6,6′-Dibromoindigo)

**DOI:** 10.3390/molecules15085561

**Published:** 2010-08-12

**Authors:** Joel L. Wolk, Aryeh A. Frimer

**Affiliations:** Department of Chemistry, Bar-Ilan University, Ramat-Gan 52900, Israel

**Keywords:** 6,6’-dibromoindigo, Tyrian purple, synthesis

## Abstract

6,6’-Dibromoindigo is a major component of the historic pigment Tyrian purple, arguably the most famous dye of antiquity. Over the last century, chemists have been interested in developing practical syntheses of the compound We describe herein a new, reasonably simple and efficient synthesis of Tyrian purple which opens the way to the production of large quantities of the dye with minimal hazards and at low cost.

## 1. Introduction

**Scheme 1:**
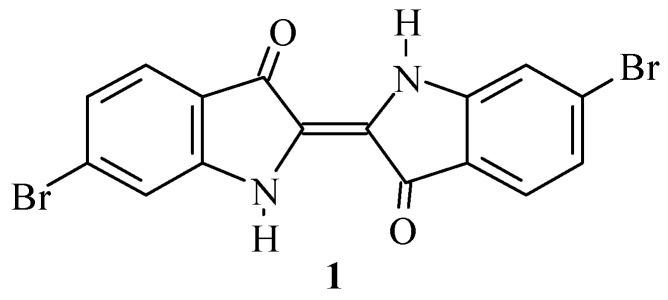
Structure of 6,6’-dibromoindigo (**1**, Tyrian purple).

6,6’-Dibromoindigo (**1**, Scheme 1) is a major component of the historic pigment Tyrian purple, and is arguably the most famous dye of antiquity [1,2].

From ancient times the dye has been produced from secretions of various species of snails found off the Atlantic and Mediterranean coasts. Due to the minute amounts of dye found in the snails, the dye has always been very costly. Paul Friedländer, who in 1909 first identified the structure of the dye as 6,6′-dibromoindigo, required 12,000 *Murex brandaris* snails to produce 1.4 g of pure pigment [3].

Ever since the dye was identified, chemists have been interested in developing practical syntheses of the compound [4]. However, all known syntheses are either lengthy, inefficient, or involve expensive or hazardous starting materials or reagents. Synthetic Tyrian purple is commercially available today, but at a price nearly as high as the natural pigment. A chemical synthesis of reasonable length, cost and safety is, therefore, still a very desirable research goal. In particular, we are interested in a synthesis which is amenable to production of relatively large amounts of the compound in an undergraduate student laboratory. This paper describes our efforts in developing a new, more attractive synthesis of Tyrian purple.

## 2. Results and Discussion

### 2.1. Preparation of Tyrian purple from 4-bromo-2-nitrobenzaldehyde

**Scheme 2:**
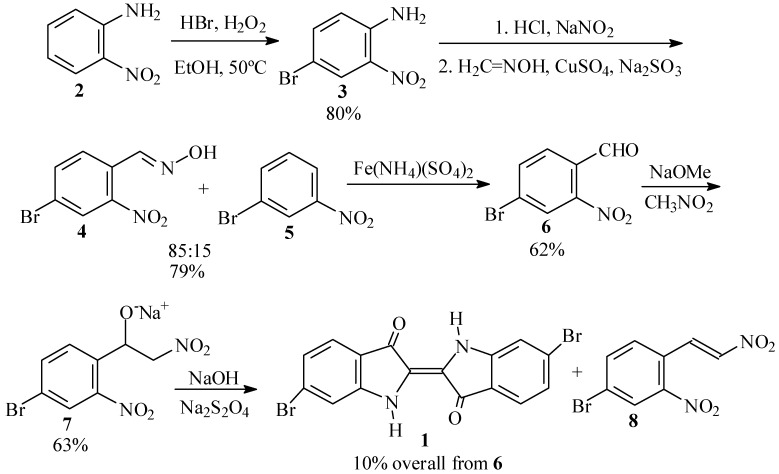
First synthetic scheme for the preparation of dibromoindigo (**1**).

Our initial efforts toward synthesis of Tyrian purple (**1**) centered around use of 4-bromo-2-nitrobenzaldehyde (**6**) as the key intermediate (Scheme 2). 

The traditional syntheses of **6** involve the use of *p*-toluidine [5], *o*-nitrotoluene [6], or their derivatives as starting materials or intermediates. Due to the toxicity and possible carcinogenicity of these compounds, we initially chose the Beech synthesis [7,8] instead, starting from *o*-nitroaniline (**2**).

In the first step of this synthesis, we prepared 4-bromo-2-nitroaniline (**3**), following the procedure of Inoue and coworkers [9,10] in which an excess of HBr and H_2_O_2_ with respect to **2** was used for the bromination. In our hands, however, this led to a mixture of **3** and a dibrominated side product, which we were unable to separate. We, therefore, adopted the optimized procedure of Islam *et al.* [11] in which H_2_O_2_ is added at 50 ºC to **3** in the presence of a slight excess of HBr in ethanol. When the amount of solvent was adjusted suitably, practically pure product, in yields of up to 80%, precipitated out from the reaction mixture upon cooling.

In the second step, 4-bromo-2-nitroaniline (**3**) is converted to 4-bromo-2-nitrobenzaldehyde (**6**). To this end, we first prepared a formaldoxime solution as prescribed by Beech [12,13]. However, diazotization of **3** in aqueous HCl [12,14] presented difficulties, as was earlier reported by Frejka and Vymetal [15]. We found it necessary to first prepare a homogeneous, finely divided suspension of the aniline hydrochloride by vigorous stirring, before adding the sodium nitrite solution. Subsequent reaction of the diazonium salt of **3** with CuSO_4_, Na_2_SO_3_ and formaldoxime in an acetate buffer gave a 79% crude yield of benzaldoxime **4**, contaminated with about 15% *m*-bromonitrobenzene (**5**). Similar formation of reduced byproduct in this reaction has been previously reported by Woodward and coworkers [16]. The oxime **4** was cleaved to **6** by boiling with excess ferric ammonium sulfate [5,12,17] in 62% crude yield. Other reagents for cleavage of the oxime gave inferior results. Thus, hexamethylenetetramine-bromine [18] gave 43% of crude **8** based on the crude oxime, while ozonolysis [19] gave only 35% of **6**. Reaction with buffered NaOCl [20] led largely to Hoffmann degradation and regeneration of the starting aniline **3**, while reflux with anhydrous FeSO_4_ [21] in acetonitrile gave no reaction. 

In the final step, the benzaldehyde **6** was transformed into dibromoindigo **1** by the Harley-Mason procedure [22], as applied by Voss and Gerlach [23] and by Cooksey [24]. Thus, reaction of **6** with nitromethane and sodium methoxide in methanol gave a 63% yield of the crude sodium nitrophenylethoxide salt **7**. However, in our hands this intermediate proved to be labile towards dehydration to a red side product, presumably the respective nitrostyrene (**8**). It is known that the initial β-nitroalcohols formed in the Henry reaction readily undergo dehydration under the basic reaction conditions to form nitroolefins [25]. Thus, reduction with basic sodium dithionite afforded only a 10% yield of crude **1** based on the starting benzaldehyde **6**.

As an alternative means of synthesizing benzaldehyde **6**, we attempted to apply the method of Li and Liu [26] of converting benzylic methyl groups to the respective aldehydes using potassium permanganate and triethylamine in the presence of sulfuric acid. This method was reported to give good yields of aldehydes from toluenes with electron withdrawing substituents. In our hands, however, both a model experiment on 2-nitrotoluene with this reagent, as well as experiments with 4-bromotoluene, gave no reaction.

### 2.2. Tyrian purple preparation from derivatives of 4-bromo-2-aminobenzoic acid (**11**)

**Scheme 3:**
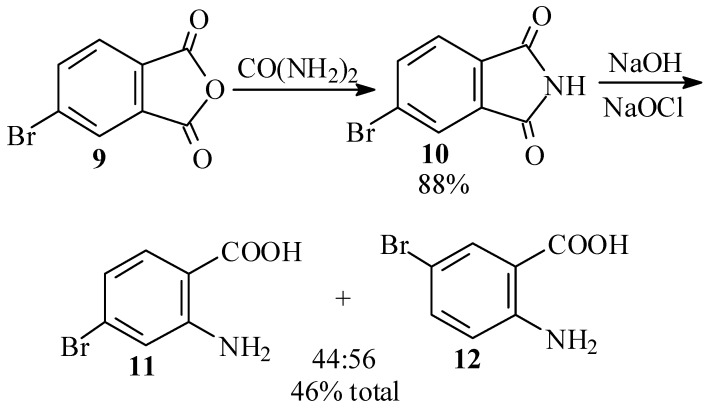
Waldmann approach [29] to 4-bromo-2-aminobenzoic acid (**11**).

In view of the tedious procedures and low yields obtained in the synthesis of **1** by way of 4-bromo-2-nitrobenzaldehyde (**6**), we next searched for an efficient synthesis of 4-bromo-2-aminobenzoic acid (**11**) as a starting material in the Friedländer procedure [3,27,28]. In our first efforts, we attempted to reproduce the Waldmann synthesis [29] of **11** by the Hofmann degradation of 4-bromophthalimide (**10**). Thus, reaction of 4-bromophthalic anhydride (**9**) with urea at 135–140 ºC gave an 88% yield of **10**. However, subsequent Hofmann degradation of **10** produced an inseparable mixture of **11** and 5-bromo-2-aminobenzoic acid (**12**), in which the latter isomer predominated slightly (Scheme 3).

**Scheme 4:**
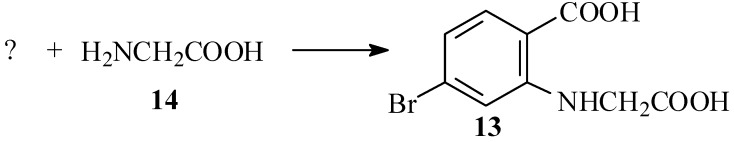
Ullmann condensation approach to phenylglycine.

We therefore turned our efforts to a search for an efficient synthesis of the substituted phenylglycine **13** by Ullmann condensation [30,31 of glycine (**14**) with a suitable substrate, as outlined in Scheme 4. 

The first substrate chosen for this reaction was 4-bromo-2-chlorobenzoic acid (**15; **see Scheme 4). This compound is available commercially, but quite expensive. We initially envisaged preparing it by chlorination of 4-bromotoluene (**16**), which has been reported to give largely 4-bromo-2-chlorotoluene (**17**) [32,33], and subsequent oxidation of the methyl group. However, prolonged standing of a solution of **16** and chlorine in Freon 113 (b.p. 47.7 ºC), a solvent similar in its properties to carbon tetrachloride used for halogenations [34], gave no reaction. 

**Scheme 5:**
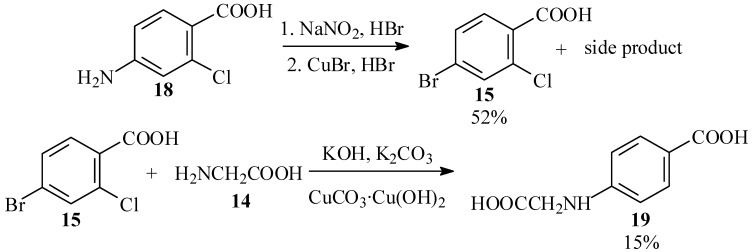
Unsuccessful approaches to phenylglycine **13**.

We, therefore, prepared the acid by Sandmeyer reaction [35,36] of 4-amino-2-chlorobenzoic acid (**18**). However, this reaction also gave an unidentified high-melting side product, and the desired product was obtained in 52% yield only after tedious chromatographic purification. Subsequent Ullmann condensation of **15** with **14** in aqueous solution, with catalysis by either copper powder [37,38] or CuCO_3_**·**Cu(OH)_2_ basic copper carbonate [39], gave modest yields of N-(4-carboxyphenyl)glycine (**19**), in which the ring bromine was replaced and the chlorine atom reduced. None of the desired product **13,** derived from displacement of the *ortho* chlorine atom, was detected (Scheme 5).

**Scheme 6:**
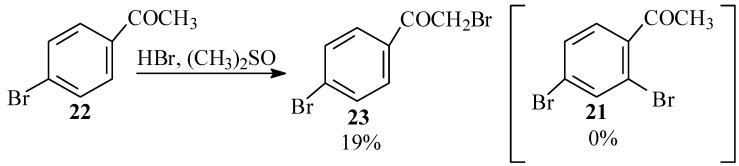
Bromination of bromacetophenone with HBr in DMSO.

Since it turned out that 4-bromo-2-chlorobenzoic acid (**18**) did not undergo Ullmann condensation to give **13**, we accordingly devoted our efforts to preparing the latter by a similar reaction of 2,4-dibromobenzoic acid (**20**). We considered preparing this compound from 2,4-dibromoacetophenone (**21**). For synthesis of the latter we first investigated the possible use of HBr and dimethylsulfoxide to effect an *ortho* bromination of 4-bromoacetophenone (**22**), analogous to the synthesis of 2-bromobenzaldehyde from benzaldehyde with this reagent as reported by Srivastava *et al.* [40] However, with this reagent, only bromination of the α carbon was observed in the product mixture yielding **23** and no 2,4-dibromoacetophenone (**21**) (Scheme 6). Furthermore, no reaction at all was observed between this reagent and either 4-bromobenzoic acid or 4-bromobenzaldehyde.

We also considered preparing **20** by oxidation of 2,4-dibromotoluene (**24**), and attempted to synthesize the latter by bromination of *p*-bromotoluene (**16**) using a “positive bromine” species generated from potassium [41,42] or sodium bromate [43,44] in a sulfuric acid medium. However, these experiments gave mixtures of products, in which ring brominated products were accompanied by large amounts of benzylic brominated compounds.

**Scheme 7:**
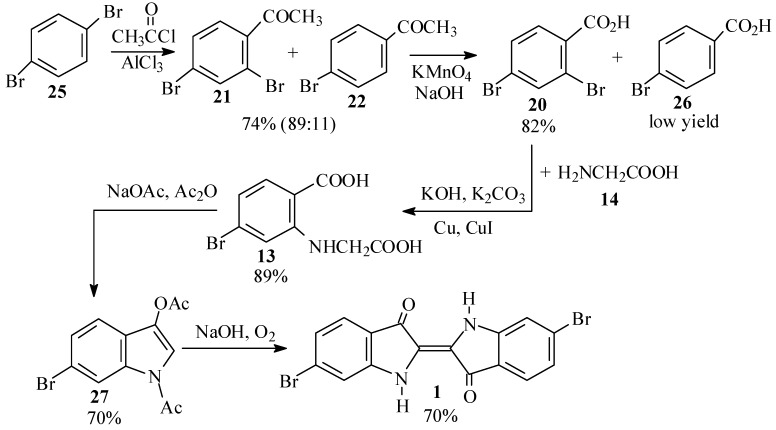
Second synthetic scheme for the preparation of dibromoindigo (**1**).

For a cleaner synthesis of 2,4-dibromoacetophenone (**21**), we therefore turned to the Friedel-Crafts acetylation of *p*-dibromobenzene (**25**) (Scheme 7), notwithstanding the conflicting reports on the products of this reaction in the literature [45,46,47,48,49,50,51].

Thus, reaction of **25** with acetyl chloride and aluminum chloride, according to the procedure of Troyanov and Dibinskaya [46], indeed gave a 64% yield of the desired product (**21**). This was proven conclusively by 2D NMR spectroscopy, in which HMBC showed a correlation between the *o,p* split ring proton and the carbonyl carbon. The product was accompanied by 20% of a fraction which was identified as mainly *p*-bromoacetophenone (**22**). When the reactants were used in the proportions as prescribed by Polívka *et al.* [50], 2,4-dibromoacetophenone **21** was isolated in 70-74% yield and was found to be 89% pure, the remainder being **22**. We also performed the acetylation using acetic anhydride [45,52,53]; however, while the product appeared to be cleaner, the isolated yields were lower (48-63%) and the crude mixture contained a little more **22** (20-25%). 

The acetyl group of the dibromoacetophenone **21** was oxidized by basic permanganate, following the procedures of Gibson and Levin [47] and later workers [45,54,55], except that instead of filtering off the precipitate of manganese dioxide after the reaction, it was decomposed by bisulfite and sulfuric acid [56]. This procedure gave good yields (68-82%) of 2,4-dibromobenzoic acid (**20**), although the crude product was contaminated by small amounts of *p*-bromobenzoic acid (**26**), and some unreacted starting material was recovered when the oxidation was run on a larger scale.

Ullmann condensation of **20** with 2 equivalents of glycine (**14**), catalyzed by a mixture of copper powder and cuprous iodide [57,58,59], proceeded vigorously at 50–60 ºC and led to high yields (up to 89% crude) of the phenylglycine **13**. Use of cuprous iodide alone, or performing the reaction at lower temperatures, led to a sluggish and incomplete reaction. The product was, however, contaminated with the impurities in the starting material, primarily *p*-bromobenzoic acid (**26**).

The phenylglycine **13** was converted to 6-bromodiacetylindoxyl (**27**) by a Claisen condensation as previously reported [28,60,61,62,63]. Workup by precipitation from the crude reaction mixture [60] gave a 70% total crude yield based on starting material, while workup on a larger scale by evaporation of the solvent [28,61] was fraught with technical difficulties and a lower isolated yield was obtained. Hydrolysis of the diacetylindoxyl **27** to Tyrian purple (**1**) was carried out with sodium hydroxide as originally reported by Friedländer and coworkers [28]. When pure **27** was used as the starting material, nearly quantitative yields of **1** were obtained, while use of crude **27** gave yields of 66 to 70%.

### 2.3. Discussion

In this work we have presented two new synthetic pathways to Tyrian purple (**1**), both of which offer significant advantages over previous approaches. Both routes are shorter and more efficient than those previously reported, and avoid the use of highly toxic or expensive reagents. The heart of the first route, Scheme 1, is a short and efficient three-step synthesis of 4-bromo-2-nitrobenzaldehyde (**6**) from *o*-nitroaniline (**2**). This involves the bromination of **2 **and subsequent conversion of the intermediate 4-bromo-2-nitroaniline (**3**) to **6** by the Beech aldehyde synthesis. However, the Beech synthesis is a rather cumbersome procedure and is attended by the formation of significant amounts of reduced product from the intermediate diazonium salt. 

Potentially attractive alternatives to this route might be found in Keinan’s regioselective nitration of *p*-bromotoluene (**16**) [64], or Otake’s regioselective bromination of *o*-nitrotoluene (**28**) in the presence of zeolites [65], to give 4-bromo-2-nitrotoluene (**29**), followed by condensation and oxidation [66,67,68,69] of the latter to give **8**. However, even with these potential innovations, this route suffers from the inherently inefficient final condensation of **6** with acetone. Even the improved yields reported by Imming [70] with the Harley-Mason procedure [22] for the condensation require strict control of reaction conditions and do not afford a better quality of product.

More attractive, in our opinion, is our five step synthesis of Tyrian purple (**1**), starting from *p*-dibromobenzene (**25; **Scheme 6). The reactions are simple, low cost, safe, high yield procedures. The first step involves the Friedel-Crafts acetylation of *p*-dibromobenzene (**25**) producing 2′,4′-dibromoacetophenone (**21**) as reported by Troyanov and Dibinskaya [46]. (This is in contrast to the reports of other workers that 4′-bromoacetophenone (**22**) or 2′,5′-dibromoacetophenone are the product of this reaction [45,47,48,49,50,51]). However, whether we used acetyl chloride or acetic anhydride as the acetylating reagent, we invariably obtained the monobromoacetophenone **22** as a side product. The latter is due to the small quantities of bromobenzene present in the reaction mixture, formed by AlCl_3_-mediated disproportionation of the starting material [71,72,73,74]. This side product is oxidized along with the main product in the subsequent step and, therefore, poses a problem in the workup. We note that performing the initial Friedel-Crafts reaction on *m*-dibromobenzene (**30**) using a preformed complex of acetyl chloride and aluminum chloride (the Perrier procedure [75,76]), would eliminate the formation of these side products. Nevertheless, the 20-fold greater cost of *m*- over *p*-dibromobenzene seems to outweigh the desire for a slightly higher yield and cleaner product.

The second step, oxidation of 2′,4′-dibromoacetophenone (**21**) to 2,4-dibromobenzoic acid (**20**), is quite straightforward. The alkaline permanganate oxidation is a standard procedure and the workup is simplified by decomposing the precipitate of manganese dioxide [56]. 

This is followed in the third step by the Ullmann condensation of 2,4-dibromobenzoic acid (**20**) with glycine (**14**) to give the bromocarboxyphenylglycine **13, **which is definitely the novel, key reaction in this synthesis. While the general condensation of 2,4-dihalobenzoic acids with glycine is listed among the claims of a recent patent [77], in practice only 2,4-dichlorobenzoic acid (**31**) has been tried and not the dibromo analogue **20** [38,39]. While the displacement of chlorine has typically been performed in aqueous systems at reflux temperatures, bromine is a much more reactive leaving group. Thus the reaction of **20** with ammonia in a biphasic water-ethyl acetate system was reported to be exothermic at room temperature, giving 4-bromoanthranilic acid (**11**) in 91% yield [78]. Another factor governing the reaction is the pH. In the condensations with 2,4-dichlorobenzoic acid (**31**) in water, it was found that the optimal results were obtained at pH 9 which minimizes reaction with the solvent and formation of chlorosalicylic acid as a side product [38]. The nature and amount of base added to the mixture also have an effect of the outcome of the Ullmann condensation [57,70,71,72,73,74,75,76,77,78,79,80,81,82]. Thus, a 95% yield of 2-carboxymethylamino-4-chlorobenzoic acid (**32**) was reported in aqueous solution when sodium carbonate was the sole base used [77]. Nonaqueous solvents have also been used in order to suppress the formation of salicylic acids as side products [39,59,80,81,82]. Variations in the procedure such as these might well be considered in efforts to optimize the reaction.

In our work the condensation of **20** with glycine was done in an aqueous system according to the original procedure for the preparation of phenylglycine-*o*-carboxylic acid (**33**) from the salt of *o*-chlorobenzoic acid (**34**) [37], except that two equivalents of potassium carbonate and a mixture of copper powder and cuprous iodide as catalysts were used [57,59,80]. Under these conditions the reaction was vigorous at 50–60 ºC and led to crude yields of **13** of up to 89%; however, these high yields often contained substantial quantities of unreacted starting material as well as *p*-bromobenzoic acid (**26**). Copper powder was essential for catalysis and the reaction did not proceed significantly in the presence of cuprous iodide alone. In view of the general consensus that a Cu(+1) species is the active catalyst [57,83,84], we may reason that the oxide layer on the metallic copper might be a more readily available source of soluble liganded Cu(+1) ions [84] than the cuprous iodide.

The fourth step involves the Claisen condensation of **13** to give the bromodiacetylindoxyl **27. **This reaction has always been performed with sodium acetate as the base in acetic anhydride as the solvent. The reaction is rapid near the boiling point of the solvent and is easily monitored by the evolution of carbon dioxide which accompanies it. The principal difficulties attend the workup and isolation of the product. In the original procedure [28,61], the solvent is removed and the residue washed with water to remove the sodium salts from the crude product. In our hands this led to a gummy residue which was difficult to work with, and to reduced yields. In view of the report that longer heating increases tar formation [61], it seems advisable to isolate the product by precipitation, either by cooling the reaction mixture [60] or by adding water without evaporating the solvent [63]. Using a combination of these methods, we indeed obtained a 70% yield of crude, isolated product. We note that the 5-bromo isomer has been prepared in 75% yield using a short reaction time and an aqueous workup [85,86]; the 6-chloro analogue has been prepared in about 50% yield after precipitating the product from the crude reaction mixture by cooling [87].

The final step involves the hydrolysis and oxidation of the diacetylindoxyl **27, **which gave very high yields of Tyrian purple (**1**) when pure starting material was used. Due to the low solubility of the product, it cannot be extracted and is therefore usually purified either by recrystallization [24,28] or by washing with several solvents [23,29]. We washed the product as did Voss and Gerlach [23], and found that only negligible amounts of product are lost in the washings. However, not all the washings may be necessary since substituted indigos may also be purified by sublimation [88].

The overall yield of Tyrian purple in our five step synthesis was about 25%, based on the starting *p*-dibromobenzene (**25**), and has yet to be fully optimized. Although this yield is lower than that achieved by Voss and Gerlach for their synthesis starting from the same compound [23], our procedure has the advantage of not requiring special techniques such as low temperatures or strictly anhydrous conditions, and is therefore amenable for student labs and industrial production of larger quantities. Our yield is also substantially greater than that reported recently by Imming and coworkers (10%) in their optimized synthesis starting from *p*-toluidine [70], and moreover our procedure does not use highly toxic or possibly carcinogenic reagents. 

In summary, we have developed a new, reasonably simple and efficient synthesis of Tyrian purple which we believe has the potential of providing large quantities of the dye with minimal hazards and at low cost.

## 3. Experimental

### General

Chemicals and solvents were used as received unless otherwise specified. Melting points were taken on a Hoover melting point apparatus and are uncorrected. NMR spectra were taken in CDCl_3_ on a Bruker Avance/DPX 300 MHz Digital NMR Spectrometer, unless otherwise specified. Chemical shifts (δ) are given in ppm.

*4-Bromo-2-nitroaniline* (**3**) [11]. To *o*-nitroaniline (**3**, 10.0 g, 0.0724 mol) in a 500 mL R.B. flask fitted with a thermometer and magnet stirring bar was added a solution of 48% HBr (8.8 mL, 0.0773 mol) in H_2_O (42 mL), followed by additional H_2_O (48 mL). The mixture was stirred at 50–55 ºC and enough 95% EtOH (about 90 mL) was added to give a clear red solution. Then 30% H_2_O_2_ (13.5 mL, 0.136 mol) was added dropwise over 15 min through an addition funnel at 50 ºC. By the end of the addition, the mixture was cloudy and orange, and a heavy red liquid separated out and presently solidified after the addition of a few more mL of EtOH. The mixture was stirred at 50 ºC for an additional 95 min and then cooled in ice to 20 ºC and filtered. Orange crystals were obtained and washed with 3 × 50 mL H_2_O and dried overnight in air. Yield 12.41 g (0.0571 mol, 80%), m.p. 105–106 ºC (lit. [89] 112-113 ºC). ^1^H-NMR: ~6.00 (b), 6.73 (d, *J* = 9 Hz, 1H), 7.43 (dd, *J* = 2, 9 Hz, 1H), 8.27 (d, *J* = 2 Hz, 1H). The product was 99% pure **3** with up to 1% unreacted **2**. Evaporation of the mother liquors furnished 1.87 g of orange to slightly yellow solids which NMR showed to contain mostly unreacted **2** and about 10% **3**. 

*4-Bromo-2-nitrobenzaldoxime* (**4**) [12,14]. A mixture of paraformaldehyde (2.58 g, 0.086 mol), recrystallized hydroxylamine hydrochloride (6.05 g, 0.086 mol) and H_2_O (38 mL) was stirred at 100 ºC for 5 min until a clear solution of formaldoxime hydrochloride was obtained. Then, sodium acetate trihydrate (11.70 g, 0.086 mol) was added and the mixture was refluxed for 15 min. The clear solution was cooled in ice. In the meantime, 4-bromo-2-nitroaniline (**3**, 9.36 g, 0.0431 mol) was placed in a 250 mL R.B. flask and 32% HCl (17 mL) was added, followed by H_2_O (42.5 mL). The mixture was stirred vigorously by magnet for 1 h until a homogeneous yellow orange precipitate was obtained, and then cooled to -1 ºC in an ice-salt bath. A solution of sodium nitrite (3.04 g, 0.0434 mol) in 8.5 mL H_2_O was added with stirring at -2 to 2 ºC over a period of 1 h. More H_2_O was added until a thin, pale orange mixture was obtained. Stirring was continued 3 more hours at 0 to 5 ºC and the mixture was filtered with ice-water cooling to give a nearly clear yellow solution.

In the meantime, the formaldoxime hydrochloride solution was transferred to a 500 mL 3-necked R.B. flask cooled in an ice-water bath. Cupric sulfate pentahydrate (2.15 g, 0.00861 mol) and 0.17 g of anhydrous sodium sulfite (0.0013 mol) were added, followed by a solution of 27.6 g of sodium acetate trihydrate (0.203 mol) in 32.5 mL H_2_O. The resulting deep green mixture was stirred with ice-water cooling. The filtered diazonium solution was neutralized, while still cooled in ice water, with a solution of sodium acetate trihydrate (a total of 13.28 g, 0.0976 mol) in about 20 mL H_2_O, giving a reddish purple color with Congo Red paper and a pH of about 3. The neutralized solution was introduced into the stirred formaldoxime solution, at 3 to 5 ºC over a period of about 105 min, through a Pasteur pipette suspended about the middle neck of the flask and extending below the surface of the liquid. This resulted in an instantaneous evolution of gas and formation of a deep green foam. The cloudy green mixture was then stirred for an additional 2 h at 8 to 10 ºC and then allowed to come to room temperature. The mixture was acidified by the addition of a few mL of concentrated HCl until a blue color was obtained on Congo Red paper. A greenish brown fine oil settled out in the bottom of the flask. The mixture was extracted with about 200 mL of ethyl ether in several portions, and the tarry material that separated out was extracted again several times until the aqueous layer was no longer green. Evaporation of the combined ether extracts gave 16 g of a semisolid brown oil, which in turn was eluted in ether through 100 g of neutral alumina. Evaporation of the eluate gave the desired 4-bromo-2-nitrobenzaldoxime (**4, **8.25 g, 0.034 mol, 79% crude yield) as an orange solid with a strong odor of acetic acid. ^1^H-NMR: 7.77, 7.84, 8.21, 8.63. The spectrum of the crude product indicates that it also contains about 13% 1-bromo-3-nitrobenzene (**5**); ^1^H-NMR: 7.45 (dt, *J* = 0.3, 8.0, 8.0 Hz, 1H), 7.84 (ddd, *J* = 1.0, 2.0, 8.0 Hz, 1H), 8.18 (ddd, *J* = 1.0, 2.0, 8.0 Hz, 1H), 8.40 (dt, *J *= 0.3, 2.0, 2.0 Hz, 1H).

*4-Bromo-2-nitrobenzaldehyde* (**6**) [6,12,17]. Crude oxime **4 **(4.91 g, 0.020 mol) was placed in a 125 mol 3-necked R.B. flask, followed by 40.0 g of ferric ammonium sulfate dodecahydrate (0.083 mol) and 60 mL H_2_O. The brown mixture was stirred under reflux for 90 min with heating in an oil bath at 125 ºC. TLC (1:1 ether-petroleum ether) showed no starting material was left. Steam was generated by boiling water in a 1 l filter flask and introduced into the reaction flask, which was set up for steam distillation with a splash guard and heated at 135 to 150 ºC. The distillate was initially a cloudy light yellow, but subsequently solidified to a yellow solid in the condenser. The distillation was stopped after 5½ h, at which point 300 mL of distillate had been collected. After cooling, the distillate was extracted with 4 × 40 mL of ethyl ether. The aqueous distillation residue was also extracted with 2 × 40 mL of ethyl ether. TLC shows product in both ether fractions. The latter fraction was evaporated to give a brown oil (1.7 g) which was eluted through 50 g of silica gel with 1:1 ethyl ether-petroleum ether to give very impure product (1.42 g). Attempted purification of this material through the bisulfite addition compound gave no significant amount of product. The ether extract of the steam distillate yielded a semisolid yellow product (2.85 g, 0.0124 mol, 62% crude yield), which could be partially crystallized from warm ethanol. ^1^H-NMR: δ 7.85 (d, *J *= 8.3 Hz, 1H), 7.93 (ddd, *J *= 0.3, 1.8, 8.3 Hz, 1H), 8.27 (d, 1.8 Hz, 1H), 10.39 (d, 0.3 Hz, 1H).

*6,6′-Dibromoindigo (1) from 4-bromo-2-nitrobenzaldehyde* (**6**) [23,24]. The crude benzaldehyde **6** (2.85 g, 0.0124 mol) in concentrated ethanol solution was dissolved in 20 mL of absolute methanol (distilled from magnesium) in a 100 mL 3-necked R.B. flask under nitrogen. nitromethane (Aldrich, 95%, 0.82 mL, 0.015 mol) was added and the mixture was stirred at -1 to 0 ºC in an ice water-salt bath. Then a cooled solution sodium methoxide (Fluka, 95%, 0.81 g, 0.015 mol) in 10 mL of absolute methanol was added dropwise over 10 min at -2 to -1 ºC. The solution turned dark red and was stirred 30 min at -1 to 0 ºC and then 1 hr at 18.5 ºC. After about 30 min a yellow solid precipitated out, along with a small amount of a red solid which floated on the orange solution. Ether (35 mL) was added and the solid filtered and washed with more ether (35 mL). More red solids appeared in the filtrate and the reaction flask. A total of 2.44 g (0.0078 mol, 63%) of mixed red and yellow solids were obtained. These, together with the concentrated filtrate, were taken up into a solution of sodium hydroxide (1.5 g, 0.0375 mol) in 65 mL, giving a dark red brown aqueous phases and a light orange ether phase. The phases were separated and the aqueous phase extracted with 2 × 20 mL of ether. The aqueous phase was cooled to 0 ºC, diluted with 10 mL water, and 70% sodium dithionite (8.7 g, 0.035 mol) was added in small portions over 15 min at 0 to 5 ºC. The mixture turned purple and a dark precipitate began to form. The temperature gradually rose to 21 ºC. Air was bubbled through the mixture for about 30 min. The fine solids were filtered, washed with a total of about 150 mL H_2_O, followed by 3 × 40 mL of ethanol and 2 × 40 mL of ether; the washings were brown. The product (0.260 g, 0.000619 mol, 10% overall yield from benzaldehyde **6**) was collected as a fine precipitate which clung to the filter paper.

*4-Bromophthalimide* (**10**) [29]. 4-bromophthalic anhydride (**9**, 5.68 g, 0.0250 mol) and urea (1.50 g, 0.025 mol) were ground up and placed in a 100 mL R.B. flask. The mixture was heated in an oil bath at 135–140 ºC. The mixture melted and evolved gas, and after 15 min a pale yellow solid was obtained. The mixture was cooled and dissolved in 25 mL of dimethylformamide with gentle warming to give a clear yellowish solution. To this was added with stirring 60 mL of H_2_O, giving a fine white precipitate. The latter was filtered, washed with 40 mL H_2_O, and dried in a vacuum to give essentially pure 4-bromophthalimide (**10, **4.95 g, 0.0219 mol, 88% yield). ^1^H-NMR: δ 7.74 (dd, *J* = 0.5, 8.0 Hz, 1H), 7.90 (dd, *J* = 1.7, 8.0 Hz, 1H), 8.01 (dd, *J* = 0.5, 1.7 Hz, 1H).

*Hofmann degradation of 4-bromophthalimide* (**10**) [29]. The phthalimide **62** (4.95 g, 0.0219 mol) was dissolved in a solution of NaOH (6.5 g, 0.163 mol) in 23 mL H_2_O in a 250 mL 3-necked R.B. flask by heating to 85 ºC. The colorless solution was cooled with stirring in an ice bath to 1–2 ºC. Then 14.55 mL of a 1.509 M solution of NaOCl (0.0220 mol), diluted by addition of 16 mL of H_2_O, was added dropwise over a period of 15 min at 1–6 ºC. The cloudy orange yellow mixture was stirred 1 hr at 1 ºC, heated rapidly to 75–78 ºC for 5 to 8 min and then cooled again in an ice-water bath. The weakly basic, clear orange yellow solution was extracted with 20 mL of ether and acidified carefully with 32% HCl to a pH of about 3. There was a little foaming and a beige precipitate appeared. This was filtered, washed with a few 20 mL portions of H_2_O, and dried in a vacuum to give of a product which consisted of a 45:55 mixture of 4- and 5-bromo-2-aminobenzoic acids (**11 **and **12 **respectively**; **2.86 g, 0.0132 mol, 60% yield). ^1^H-NMR of 4-bromo-2-aminobenzoic acid (**11**): δ 6.79 (dd, *J *= 1.8, 8.5 Hz, 1H), 6.86 (d, *J* = 1.8 Hz, 1H), 7.76 (d, *J* = 8.5 Hz, 1H). ^1^H-NMR of 5-bromo-2-aminobenzoic acid (**12**): δ 6.58 (d, *J* = 8.7 Hz, 1H), 7.37 (dd, *J* = 2.4, 8.7 Hz, 1H), 8.03 (d, *J* = 2.4 Hz, 1H). Neither recrystallization from ethanol-H_2_O nor extraction with heptane afforded any significant separation of the isomers.

*4-Bromo-2-chlorobenzoic acid* (**15**) [35,36]. 4-amino-2-chlorobenzoic acid (**18**, Aldrich, 2.50 g, 0.0146 mol) was placed in a 250 mL 3-necked R.B. flask, followed by 48% HBr (8.5 mL, 0.073 mol) and about 110 mL of H_2_O. The mixture was heated to about 60 ºC with stirring until a clear orange solution was obtained. This was cooled with stirring in an ice-salt water bath to -3 ºC, and a white precipitate appeared. Then a solution of sodium nitrite (1.01 g, 0.0146 mol) in 6–7 mL H_2_O was added by pipette over about 30 min. The white suspension of the hydrobromide salt broke up and a yellow-orange mixture was obtained, along with some precipitate on the sides of the flask towards the end of the addition. The heterogeneous mixture was stirred at -3 ºC.

In the meantime cupric sulfate pentahydrate (4.57 g) was dissolved in 16 mL H_2_O and heated with stirring to 70 ºC. Sodium bromide (2.24 g, 0.0218 mol) was added slowly with stirring in small portions. An initially dark precipitate appeared and the solution turned green. Then an aqueous solution of NaOH (0.6 g, 0.015 mol) and sodium bisulfite (Aldrich, min. SO_2_ content 58.5%, 0.99 g, 0.00825 mol) was heated to 70 ºC and added to the cupric bromide solution. A thin white precipitate appeared and the supernatant was light blue. Three more portions of sodium bisulfite (0.1 g in 1 mL H_2_O) were added, but the blue color remained. The mixture was cooled in an ice-water bath, the solid allowed to settle, the supernatant decanted and the solid washed with a few portions of 10 mL H_2_O. The solid was then dissolved in a total of 14 mL 48% HBr and the purple solution introduced into a 500 mL R. B. flask.

The diazonium salt slurry was then introduced into the CuBr-HBr solution beneath the surface of the liquid, under cooling with an ice-salt bath and stirring over a period of 1½ h. Much frothing occurred and the mixture turned a greenish brown. The mixture was stirred another 1 h at RT and 1½ h at 55−60 ºC. The mixture was cooled, filtered, and the soft tan solid product washed with 2 × 5 mL of 48% HBr each diluted with 20 mL H_2_O, followed by 2 × 25 mL H_2_O. The crude product (7.53 g) was dissolved in a solution of KOH (3.5 g) in 50 mL H_2_O, giving a deep red brown mixture. This was filtered and the filtrate acidified with 32% HCl to pH 1, giving a beige solid which was filtered off, washed with 20 mL H_2_O and dried in vacuum to give crude product (2.92 g, 0.0124 mol, 85% yield) which melted partly at 162 ºC but contains another, high melting component. ^1^H NMR also showed a mixture of two compounds, and comparison with a commercial sample showed that the predominant, more soluble fraction in H_2_O is the desired 4-bromo-2-chlorobenzoic acid (**15**). However, purification by extraction was not feasible. Purified product (1.773 g, 0.00752 mol, 52% yield) was purified by column chromatography on 100 g of silica gel, using a 2:1:0.06 mixture of ethyl acetate/petroleum ether/acetic acid. Mp: 162−164 ºC (lit. [32] 166−167 ºC). ^1^H NMR (CDCl_3_/DMSO-*d*^6^): δ 7.46 (dd, *J* = 1.9, 8.4 Hz, 1H), 7.62 (d, *J* = 1.9 Hz, 1H), 7.79 (d, *J* = 8.4 Hz, 1H). ^13^C NMR (CDCl_3_/DMSO-*d*^6^): δ 125.41, 129.57, 130.05, 132.46, 132.98, 133.61.

*2′,4′-Dibromoacetophenone* (**21**) [46]. Aluminum chloride (10.0 g, 0.075 mol) was placed in a R.B. flask equipped with a condenser and silicone oil gas bubbler outlet leading to a trap filled with a 10% aqueous NaOH solution. *p*-Dibromobenzene (**25**, 7.08 g, 0.030 mol) was added and the mixture was gently agitated. Then acetyl chloride (3.2 mL, 3.53 g, 0.045 mol) was added by pipette, leading to some fuming was noticed. The reaction vessel was immersed into an oil bath preheated to 93 ºC. The mixture melted and vigorous evolution of gas ensued for about 15 min. The mixture was stirred for 2½ h at 90−92 ºC, by which time it had gradually turned brown and TLC (1:6: ethyl acetate/petroleum ether) showed no starting material and two more slowly moving products. After 3 hr the mixture was poured with stirring into a mixture of 60 g of ice and 25 mL of 32% HCl. A creamy yellow solid separated and the liquid phase was yellow. The mixture was extracted with 5 × 25 mL of ethyl ether and the combined ether phase was washed with 2 × 10 mL H_2_O, 10 mL of 10% sodium carbonate solution, 2 × 7 mL H_2_O and finally 5 mL of saturated NaCl solution; the last washings were neutral. The yellow-orange organic layer was dried with sodium sulfate and magnesium sulfate, filtered, and the solvent distilled off to give a brown oil (8.32 g). NMR showed the crude product to be 89% pure 2′,4′-dibromoacetophenone (**21**), with the remainder being mainly p-bromoacetophenone (**22**). Distillation of the crude product at 0.02 Torr yielded a forerun at 76−81 ºC, followed by a main fraction (5.80 g, 0.0209 mol, 70%) at 81−84 ºC/0.02 Torr. ^1^H NMR (acetone-*d*^6^): δ 2.60 (s, 3H), 7.55 (dd, *J* = 0.2, 8.4 Hz, 1H), 7.63 (dd, *J* = 1.8, 8.4 Hz, 1H), 7.83 (dd, *J* = 0.2, 1.8 Hz, 1H). ^13^C NMR (acetone-*d*^6^): δ 30.06, 119.60, 125.24, 131.16, 131.43, 136.43, 140.76, 199.60. The aforementioned forerun crystallized partially and proved to be mainly p-bromoacetophenone (**22, **0.988 g, 0.00496 mol). ^1^H NMR (CDCl_3_): δ 2.59 (s, 3H), 7.61 (m, 2H), 7.82 (m, 2H). When the reaction was performed on a 3-fold scale of *p*-dibromobenzene (23.60 g, 0.100 mol scale) at 65−75 ºC, the crude product contained about 80% of **21. **Distillation gave a main cut of 20.71 g (74% yield) at 87−98.5 ºC/0.07 Torr which contained 12% of impurities as *p*-bromoacetophenone as determined by NMR. ^1^H NMR (CDCl_3_, 600 MHz): δ 2.62 (s, 3H), 7.37 (d, *J* = 8.3 Hz, 1H), 7.52 (dd, *J *= 1.8, 8.3 Hz, 1H), 7.80 (d, *J *= 1.8 Hz, 1H). ^13^C NMR (CDCl_3_, 600 MHz): δ 30.20, 119.89, 125.41, 130.21, 130.71, 136.31, 139.96, 200.05. 2D NMR: HMQC correlations: 7.37–130.21; 7.52–130.71; 7.80–136.31. HMBC correlations: 2.62–30.20, 139.96, 200.05; 7.37–119.89, 125.41, 130.71, 136.41, 200.05; 7.52–119.89, 125.41, 130.21, 136.31, 139.96; 7.80–119.89, 125.41, 130.71, 139.96.

*2,4-Dibromobenzoic acid* (**20**) [47]. Distilled 2′,4′-dibromoacetophenone (**21**, 4.60 g, 0.0166 mol) was placed in a 500 mL R.B. flask fitted with a magnetic stirrer. A solution of NaOH (1.05 g, 0.0263 mol) in 10.5 mL H_2_O was added, followed by a solution potassium permanganate (7.50 g, 0.0475 mol) in 260 mL H_2_O. The mixture was stirred under reflux using an oil bath heated to 143−155 ºC for 5 h, at which time the permanganate color from the aqueous phase had disappeared. The mixture was allowed to cool, acidified with a solution of 5 mL of concentrated sulfuric acid in 10 mL H_2_O, and the black precipitate of manganese dioxide decomposed with sodium bisulfite (Fluka, 64% SO_2_, a total of 6.7 g). The mixture was adjusted to pH 7 by addition of 6 M NaOH followed by 6 M H_2_SO_4_ and then extracted with a total of 190 mL of ethyl ether in 4 portions. The aqueous layer was then acidified by adding small aliquots of 6 M H_2_SO_4_ with stirring, and a thick white precipitate was obtained. This was filtered off, washed with 2 × 20 mL H_2_O, and dried in vacuo to give of the desired product (3.35 g) softening at 145 ºC and melting at 162−165 ºC, (lit. [90] 169 ºC). The latter proved by NMR to be fairly pure 2,4-dibromobenzoic acid (**20**) containing a few percent of *p*-bromobenzoic acid (**26**). ^1^H NMR (acetone-*d*^6^): δ 7.71 (dd, *J* = 1.9, 8.3 Hz, 1H), 7.84 (d, *J* = 8.3 Hz, 1H), 7.96 (d, 1.9 Hz, 1H). From the mother liquors of the aqueous layer an additional 0.49 g of crystalline material was obtained that melted mostly at 145 ºC and was identified by NMR as mainly 2,4-dibromobenzoic acid (**20**). Total yield was 82% (3.81 g, 0.136 mol).

*4-Bromo-2-[(N-carboxymethyl)amino]benzoic acid* (**13**) [57,58,59]. 2,4-Dibromobenzoic acid (**67**, 2.80 g, 0.010 mol) was placed in a 100 mL R.B. flask and a solution of 85% KOH (0.66 g, 0.010 mol) in 2.5 mL H_2_O was added with stirring. Heat was generated and a homogenous slurry was obtained. The glycine (1.50 g, 0.020 mol) was added, followed by copper powder (0.066 g, 00104 mol) and cuprous iodide (0.074 g, 0.000389 mol). The grayish slurry was immersed into an oil bath at 60 ºC and potassium carbonate (1.38 g, 0.010 mol) was added. Frothing occurred and a blue color and a thick precipitate appeared, and 7.5 mL H_2_O was added to a total of 10 mL. The grayish blue green mixture was stirred for a total of 1½ h, after which TLC (2:1:0.06: ethyl acetate/petroleum ether – acetic acid) showed no starting material remained. The mixture was diluted with 30 mL H_2_O and acidified to pH 1 with 3–5 mL of 32% HCl. Foaming occurred and a thick violet precipitate appeared. The mixture was extracted with 130 mL of ethyl ether in 5 portions, and the combined extracts were washed with a little H_2_O, 4 × 5 mL of 5% aqueous disodium EDTA, 5 mL of saturated aqueous NaCl and dried with magnesium sulfate. Evaporation of the solvent at 35 ºC gave crude product (2.21 g, 0.00806 mol, 81% yield) as an off-white solid. ^1^H NMR (DMSO-*d*^6^): δ 4.04 (s, 2H), 6.76 (dd, *J* = 1.9, 8.4 Hz, 1H), 6.79 (d, *J* = 1.9 Hz, 1H), 7.71 (d, *J* = 8.4 Hz, 1H), 8.22 (b, 1H), ~12.9 (b).

*6-Bromo-N,O-diacetylindoxyl*** (27) ** [28,60,61,62,63]. Bromocarboxyphenylglycine **13** (2.15 g, 0.00785 mol) was dissolved in 20 mL of redistilled acetic anhydride in a 50 mL R.B. flask with gentle warming. Then anhydrous sodium acetate (0.81 g, 0.010 mol) was added and the flask was immersed into an oil bath preheated to 135−137 ºC. The solids readily dissolved and the mixture turned a cloudy yellow brown with evolution of gas. After 10–15 min the cloudiness disappeared and evolution of gas stopped. TLC (2:1:0.06: ethyl acetate/petroleum ether/acetic acid) showed that no starting material remained. The mixture was allowed to cool to room temperature and a yellowish precipitate appeared. This was filtered off, washed with a solution of sodium acetate (0.6 g) in 5 mL of acetic acid, and was found to dissolve in 15 mL of ice cold H_2_O. The combined organic layers were cooled in ice, a small amount of crystalline solid material filtered off, and the filtrate combined with the cooled aqueous layer and refrigerated. A yellow solid separated out which was then filtered, washed with 4 × 5 mL H_2_O and then dried in vacuo to give 1.11 g of product which was shown by NMR to be quite pure 6-bromodiacetylindoxyl (**27**). ^1^H NMR: δ 2.38 (s, 3H), 2.60 (s, 3H), 7.40 (dd, *J* = 0.6, 8.4 Hz, 1H), 7.44 (dd, *J* = 1.5, 8.4 Hz, 1H), 7.71 (s, 1H), 8.70 (b, 1H) The filtrate was evaporated to dryness in vacuo and the yellow brown solid residue was worked up similarly. The aqueous layer was extracted with ether, giving a total of 0.51 g additional product which was less pure than the first fraction. The total yield of **27 **was 70% (1.62 g, 0,00547 mol).

*6,6′-Dibromoindigo (1) from 6-bromodiacetylindoxyl* **(27)**. Pure 6-bromodiacetylindoxyl (**27**, 1.11 g, 0.00372 mol) was placed in a 50 mL R.B. flask and a solution NaOH (2.0 g, 0.050 mol) in 25 mL H_2_O was added. The mixture was immersed with stirring into an oil bath preheated to 130 ºC. The mixture turned brown and a mauve purple color began to appear almost instantly. The mixture was stirred under reflux for 30 min and then at room temperature overnight. It was then divided into 4 portions, and each was centrifuged in a 10 or 15 mL cuvette. The solids were then washed twice with hot water, twice with ethanol and twice with ether, centrifuging the solids each time. The ethanol and ether washings were reddish to bright rose violet. The residual purple solids were dried in vacuo to give a 93%yield dibromoindigo (**1, **0.725 g, 0.00173 mol).

## 4. Conclusions

From ancient times, the dye of royalty Tyrian purple has been produced from secretions of various species of snails found off the Atlantic and Mediterranean coasts. Ever since its chemical identification a century ago, chemists have been interested in developing practical syntheses of the compound. However, all known syntheses are either lengthy, inefficient, or involve expensive or hazardous starting materials or reagents. Synthetic Tyrian purple is commercially available today, but at a price nearly as high as the natural pigment. 

We have described in this paper a new, reasonably simple and efficient synthesis of Tyrian purple which we believe has the potential of providing large quantities of the dye with minimal hazards and at low cost. The overall yield of Tyrian purple in our five step synthesis was about 25%, based on the starting *p*-dibromobenzene (**25**), and has yet to be fully optimized. Although this yield is lower than that achieved by Voss and Gerlach for their synthesis starting from the same compound [23], our procedure has the advantage of not requiring special techniques such as low temperatures or strictly anhydrous conditions; nor does our procedure use highly toxic or possibly carcinogenic reagents. Therefore, this procedure is amenable for student labs and industrial production of larger quantities. 
